# A First Insight into *Pycnoporus sanguineus* BAFC 2126 Transcriptome

**DOI:** 10.1371/journal.pone.0081033

**Published:** 2013-12-02

**Authors:** Cristian O. Rohr, Laura N. Levin, Alejandro N. Mentaberry, Sonia A. Wirth

**Affiliations:** 1 Instituto de Ecología, Genética y Evolución, Consejo Nacional de Investigaciones Científicas y Técnicas, Ciudad de Buenos Aires, Buenos Aires, Argentina; 2 Laboratorio de Micología Experimental, Departamento de Biodiversidad y Biología Experimental, Universidad de Buenos Aires, Ciudad de Buenos Aires, Buenos Aires, Argentina; 3 Laboratorio de Agrobiotecnología, Universidad de Buenos Aires, Ciudad de Buenos Aires, Buenos Aires, Argentina; AIT Austrian Institute of Technology GmbH, Austria

## Abstract

Fungi of the genus *Pycnoporus* are white-rot basidiomycetes widely studied because of their ability to synthesize high added-value compounds and enzymes of industrial interest. Here we report the sequencing, assembly and analysis of the transcriptome of *Pycnoporus sanguineus* BAFC 2126 grown at stationary phase, in media supplemented with copper sulfate. Using the 454 pyrosequencing platform we obtained a total of 226,336 reads (88,779,843 bases) that were filtered and *de novo* assembled to generate a reference transcriptome of 7,303 transcripts. Putative functions were assigned for 4,732 transcripts by searching similarities of six-frame translated sequences against a customized protein database and by the presence of conserved protein domains. Through the analysis of translated sequences we identified transcripts encoding 178 putative carbohydrate active enzymes, including representatives of 15 families with roles in lignocellulose degradation. Furthermore, we found many transcripts encoding enzymes related to lignin hydrolysis and modification, including laccases and peroxidases, as well as GMC oxidoreductases, copper radical oxidases and other enzymes involved in the generation of extracellular hydrogen peroxide and iron homeostasis. Finally, we identified the transcripts encoding all of the enzymes involved in terpenoid backbone biosynthesis pathway, various terpene synthases related to the biosynthesis of sesquiterpenoids and triterpenoids precursors, and also cytochrome P450 monooxygenases, glutathione S-transferases and epoxide hydrolases with potential functions in the biodegradation of xenobiotics and the enantioselective biosynthesis of biologically active drugs. To our knowledge this is the first report of a transcriptome of genus *Pycnoporus* and a resource for future molecular studies in *P. sanguineus*.

## Introduction

Plant cell walls are mainly composed of cellulose, hemicellulose and lignin and constitute the most abundant source of organic carbon on Earth. Though lignocellulose is highly recalcitrant to degradation, there are many organisms capable of hydrolyzing it, including members of the intestinal microflora of ruminants and the insects and fungi responsible for wood decay. Among the latter, the basidiomycetes causing white rot are particularly effective in using the lignocellulose of plant cell walls as carbon source through the synthesis of a considerable number of hydrolytic enzymes, including cellulases, hemicellulases, pectinases and also lignin-modifying enzymes and other accessory enzymes, which can be employed in a wide range of industrial processes [1]. One of the most promising applications of these enzymes is their use to process plant biomass into fermentable sugars for the production of second-generation biofuels. Additionally, many lignocellulolytic enzymes are used in the bleaching of paper and pulp, the processing of food and textiles, as additives for soaps and detergents and also as animal feed supplements [2]–[4]. Furthermore, several lignin-modifying enzymes are non-specific phenol oxidases and peroxidases capable of oxidizing xenobiotics such as nitroaminotoluens, chlorophenols, polycyclic aromatic hydrocarbons, organophosphates, aromatic phenols and textile dyes, thus showing large potential as bioremediation agents [5]–[7]. Meeting of these demands requires bioprospecting of new enzyme sources, development of more stable biocatalysts through protein engineering and availability of new systems for massive enzyme production.

High-throughput sequencing facilitated the access to genomic and transcriptomic data and accelerated the process of enzyme discovering. Since the sequencing of the first white-rot fungus genome, *Phanerochaete chrysosporium* [8], an increasing number of genomes and transcriptomes of wood decay basidiomycetes have been reported. Except for the genomes of *Schizophyllum commune* [9], *Postia placenta* [10] and *Serpula lacrymans* [11] most of the remaining were reported in 2012, including those of *Ceriporiopsis subvermispora* [12], *Ganoderma lucidum* [13] *Fibroporia radiculosa* [14], *Phanerochaete carnosa* [15] *Heterobasidion irregulare* [16] *Auricularia delicata*, *Coniophora puteana*, *Dacryopinax* sp, *Dichomitus squalens*, *Fomitiporia mediterranea*, *Fomitopsis pinicola*, *Gloeophyllum trabeum*, *Trametes versicolor*, *Punctularia strigosozonata*, *Stereum hirsutum* and *Wolfiporia cocos* [17].

Fungi of the genus *Pycnoporus* are basidiomycetes that cause wood decay by white rot. There are four widely distributed species, *Pycnoporus cinnabarinus*, *Pycnoporus puniceus*, *Pycnoporus sanguineus* and *Pycnoporus coccineus*. Strains of *Pycnoporus* were described by their ability to synthesize compounds of high added-value, including flavors, antioxidants, antibiotics and antivirals [18]–[22] and as efficient producers of laccases and other enzymes of industrial interest [23]–[31]. Although many of these enzymes -showing high thermal stability, broad pH range, and potential in biotechnological applications-, have been purified and characterized, there is a lack of exhaustive molecular studies and no genomic or transcriptomic data is so far available for this genus.

The ability of *P. sanguineus* BAFC 2126, to selectively delignify loblolly pine (*Pinus taeda*) chips was already proven [32]. Fungal pretreatment caused changes in wood chemical composition as well as in physical structure. Experimental results showed that *P. sanguineus* was able to reduce lignin content in 11% in 14 days of treatment, and that *P. taeda* wood suffered notable structural changes of lignin and hemicelluloses, as revealed from ^13^C CP-MAS NMR spectra. An increase of 15% in porosity of decayed wood confirmed physical changes due to fungal attack. Thus, this strain is potentially a candidate for use in softwoods biopulping processes.

In this work we sequenced and analyzed the transcriptome of *P. sanguineus* BAFC 2126. Since it was reported that the addition of Cu^2+^ in culture media induces the transcription of laccase genes in white-rot fungi [33], [34] and also the expression of other enzymes such as glyoxal oxidase and manganese peroxidase [35], we evaluated the transcriptome of *P. sanguineus* growing in media supplemented with copper sulfate. Our results provide the first reference transcriptome of the genus *Pycnoporus* and a resource for future molecular studies in *P. sanguineus*.

## Materials and Methods

### Organism and culture conditions

*P. sanguineus* strain BAFC 2126 (BAFC: Mycological Culture Collection of the Department of Biological Sciences, Faculty of Exact and Natural Sciences, University of Buenos Aires) (Polyporaceae, Aphyllophorales, Basidiomycetes) was used in this study. Stock cultures were maintained on malt extract agar slants at 4°C. Medium for fungal culture (GA medium) contained 20 g glucose, 3 g asparagine monohydrate, 0.5 g MgSO_4_ ·7H_2_O, 0.5 g KH_2_PO_4_, 0.6 g K_2_HPO_4_, 0.09 mg MnCl_2_ ·4H_2_O, 0.07 mg H_3_BO_3_, 0.02 mg Na_2_MoO_4_ ·H_2_O, 1 mg FeCl_3_, 3.5 mg ZnCl_2_, 0.1 mg thiamine hydrochloride in 1 L of distilled water and supplemented with 1 mM CuSO_4_. Initial pH of the medium was adjusted to 6.5 with 1 N NaOH. Erlenmeyer flasks (500 ml size) containing 50 ml of medium were inoculated with four 25-mm^2^ surface agar plugs from a 7-day-old culture grown on malt agar (1.3% malt extract, 1% glucose, 2% agar). Incubation was carried out statically at 28 ±1°C. Cultures were harvested at stationary phase at day 21.

### RNA extraction, cDNA synthesis and 454 pyrosequencing

Fungal mycelium was filtered and immediately ground into fine powder using liquid nitrogen. Total RNA was extracted using the RNAzol RT reagent (Molecular Research Center Inc., Cincinnati, USA) according to the manufactureŕs instructions. The quantity of RNA was estimated in a Nanodrop ND-1000 spectrophotometer (Nanodrop Technologies) and RNA quality was determined by formaldehyde RNA gel electrophoresis. Poly (A) RNA was purified from total RNA using Dynabeads oligo (dT) magnetic beads (Invitrogen Life Technologies, Carlsbad, USA) and mRNA was broken into fragments of 50 to 2000 nucleotides by treatment with RNA fragmentation buffer (0.1 M Tris-HCl, pH 7.0 and 0.1 M ZnCl_2_) and heating at 70°C for 30 s. Fragmented mRNA quality was assessed by Agilent 2100 Bioanalyzer (Agilent Technologies, CA, USA). Short mRNA sequences were used for double strand cDNA synthesis using the cDNA Synthesis System Kit (Roche) and random primers, followed by purification by QIAQuick PCR Purification kit (Qiagen Inc., CA, USA). The final cDNA library was constructed using the GS FLX Titanium Rapid Library Preparation Kit (Roche). Sequencing was carried out using the Roche 454 GS FLX pyrosequencing platform (INDEAR/CONICET, Rosario, Argentina).

### Assembly and functional annotation

Reads were assembled using the Newbler v2.6 software (Roche). Similarities BLAST search for the transcripts were done against the NCBI non-redundant (nr) (ftp://ftp.ncbi.nih.gov/blast/db/FASTA/nr.gz) and UniProt (http://www.uniprot.org/) protein databases using BLASTx algorithm with a cutoff e-value of 10^−5^. House-made perl scripts were used to parse the results. Blast2GO suite was used to annotate the transcripts with Gene Ontology (GO) information [36]. KEGG pathways were annotated using KEGG Automatic Annotation Server (KAAS) [37]. Enzyme commission numbers (EC number; http://enzyme.expasy.org/) were assigned from the blast top hits.

Best open reading frames (ORFs) were predicted using OrfPredictor and blasted versus the NCBI nr database. ORFs were analyzed using SignalP for the presence and location of signal peptide cleavage sites and TargetP to predict the subcellular location. HMMSEARCH from the HMMER package was used to scan the transcripts against the PFAM and TIGRFAM protein domain databases.

Carbohydrate Active Enzymes family prediction was done using the CAZYmes Analysis Toolkit (CAT) [38] and manually curated by searching homologies to previously annotated CAZymes in the NCBI nr protein database.

### Data availability

The raw sequencing data of *P. sanguineus* was submitted to the NCBI Sequence Read Archive under the accession number SRA082106. The Transcriptome Shotgun Assembly project has been deposited at DDBJ/EMBL/GenBank under the accession GAKI00000000. The version described in this paper is the first version, GAKI01000000, and consists of sequences GAKI01000001-GAKI01007303.

## Results

### Sequencing and *de novo* transcriptome assembly

The cDNA libraries were synthesized using RNA extracted from 3-weeks-old stationary-phase *P. sanguineus* cultures grown in presence of Cu^2+^, and sequenced using a Roche 454 GS FLX pyrosequencing platform. The shotgun sequencing yielded 226,336 raw reads (88,779,843 bases) with an average length of 395.45 ± 148.24 bp that were filtered for adaptor sequences, primers and trimming of low-quality bases. The sequences were *de novo* assembled using the Newbler software v2.6 (Roche), resulting in 7,986 contigs. The overlapping contigs were assembled in 7,952 isotigs (equivalent to unique RNA transcripts) (Table 1).

**Table 1:** Summary of *P. sanguineus* transcriptome assembly.

Number of contigs	7,986
Total size of contigs (bp)	5,802,922
Longest contig (bp)	4,764
Number of contigs > 1K nt	1,513 (18.9%)
Mean contig size (bp)	727
Median contig size (bp)	609
N50 contig length (bp)	822
L50 contig count	2,330

After assembly, some sequences contained high similarity causing over-representation for transcript count. To remove spurious isoforms we run cd-hit-454 with 95% similarity cut off [39]. All the transcripts with length lower than 200 bp were also removed. After filtering, a reference transcriptome of 7,303 transcripts was generated (Table S1).

The assembly was also validated by testing the homology to the *Pycnoporus* genus sequences already annotated in the NCBI database (encoding a total of 135 proteins). To this end, a tBLASTn algorithm with an E-value cut off threshold of 10^−10^ was run against our assembled transcripts (Table S2). Significant hits (>77% identity) were observed to 116 redundant sequences (85.9%), including transcripts for beta-tubulin, translation elongation factor 1-alpha, RNA polymerase II subunits, glyceraldehyde-3-phosphate dehydrogenase, laccase, manganese peroxidase and lignin peroxidase. Conversely, no hits were observed for tyrosinase (GenBank AAX46018 and AAX44240), cellobiose dehydrogenase (GenBank AAC32197) and mitochondrial ATP synthase subunit 6 (GenBank ACA63368).

### Functional annotation of *P. sanguineus* transcriptome

**Figure 1:**
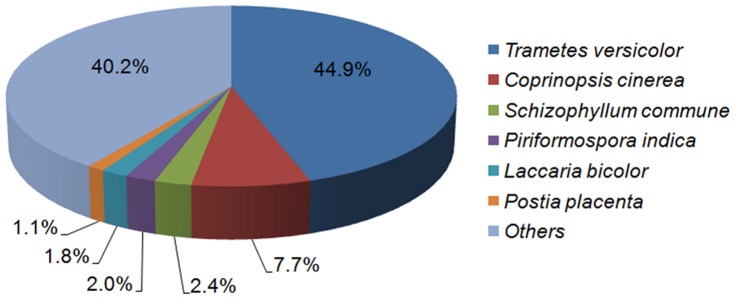
Top hits distribution of BLASTx against custom database.

Potential protein-coding transcripts were identified employing the BLASTx algorithm with a cutoff E-value threshold of 10^−5^ against the NCBI nr peptide database. This search yielded 6,109 transcripts (83.6%) similar to known proteins or conserved hypothetical proteins. We also performed a blast against the dbEST database of NCBI using BLASTn with an E-value cutoff of 10^−5^, obtaining a total of 5,734 transcripts (78.5%) with a match. From transcripts no matching against the NCBI nr database, 320 (4.4%) did match against the dbEST database and from the remaining transcripts, 549 (7.5%) had ORFs > =  80 amino acids that could represent putative *P. sanguineus*-specific protein-coding genes. As over half of the hits versus the NCBI nr database, are predicted or hypothetical proteins, we decided to create a customized database, including the sequences corresponding to basidiomycetes from the UniProt database (Swiss-Prot and TrEMBL) and the *T. versicolor* and *P. chrysosporium* sequences present in the NCBI database. A BLASTx search was performed against this database with a cutoff E-value threshold of 10^−5^, and a house-made Perl script was used to filter the hits, leaving only those that did not contain the words “hypothetical”, “predicted” or “uncharacterized” (Table S1). Top blast hits belong to *T. versicolor* (44.9%), followed by *Coprinopsis cinerea* (7.7%) and *S. commune* (2.4%) (Figure 1).

The high similarity found between *T. versicolor* and *P. sanguineus* can be mainly explained by the fact that they are closely related species. *Trametes* and *Pycnoporus* were grouped in one clade in previous studies examining DNA sequences of genomic and mitochondrial ribosomal DNA [40], [41]. Although the only morphological feature delimiting these genera is the conspicuous bright reddish-orange color of the basidiocarp, the black KOH reaction on all parts of the basidiomes clearly separates *Pycnoporus* from *Trametes* [42], [43]. Phylogenetic analysis based on the combination of ITS and RPB2 sequences confirmed the close relationship between the two genera; nevertheless the *Trametes* clade was proposed to be divided in four branches: 1) *Trametes*, corresponding to the species with pubescent/hirsute upper surface, including most temperate species fitting the traditional definition of the genus, in addition to “*Lenzites*” *betulinus* and “*Coriolopsis*” *polyzona*; 2) *Pycnoporus*, including species with red basidiomes, blackening with KOH; 3) *Artolenzites*, including the tropical “*Lenzites*” *elegans*; 4) *Leiotrametes* gen. nov., comprising three tropical species: “*Trametes*” *menziesii*, *Trametes lactinea*, “*Leiotrametes* sp.”[44]. In a large phylogenic study of *Pycnoporus*, Lesage-Meessen *et al*. [45] clearly separated four species within the genus (*P. sanguineus*, *P. puniceus*, *P. coccineus* and *P. cinnabarinus*) and defined the genetic intraspecific variability of each of them according to their geographic distribution.

**Figure 2:**
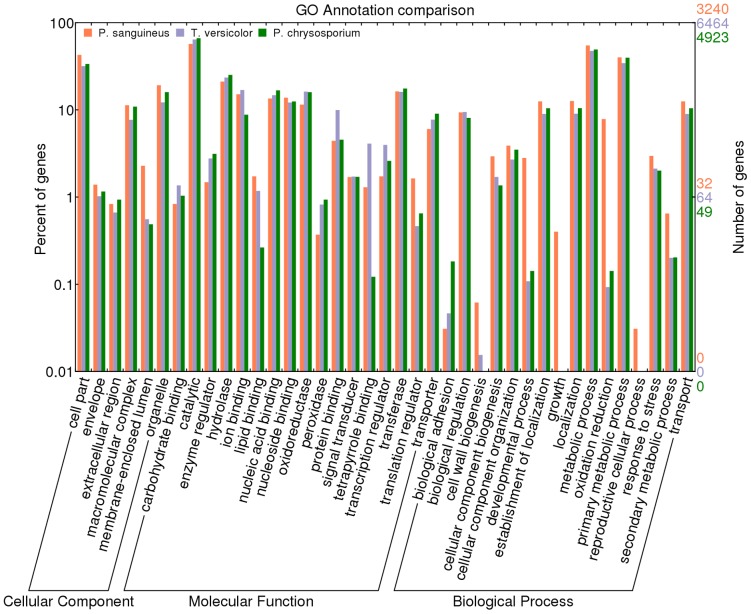
Comparative GO annotation of the *P. sanguineus* transcriptome. GO terms assigned to 3,240 *P. sanguineus* transcripts were grouped into GO slim terms (x-axis) in the three main categories (Biological process, Cellular component, Molecular function) and compared to annotations for *T. versicolor* (6,464 genes) and *P. chrysosporium* (4,923 genes). The percentage of genes represents the number of genes of each category in reference to total genes.

Gene ontology terms were annotated using Blast2GO, which assigned 10,114 GO terms to 3,240 transcripts (44.4%) (Table S3). Most abundant GO slim terms for molecular functions include catalytic and hydrolase activities, ion binding, nucleotide binding, oxidoreductase activity and transferase activity, reflecting the ability of *P. sanguineus* to degrade diverse organic compounds through the production of hydrolytic enzymes and redox processes. The WEGO server [46] was used to compare the annotations from *P. sanguineus* to two related organisms, *T. versicolor* and *P. chrysosporium.* An overview analysis showed a similar distribution of transcripts among different functional categories, as it was expected due to the taxonomic proximity between these three species (Figure 2).

EC numbers were assigned to 1,400 (19.2%) transcripts from the top BLAST hits (Table S1). The EC number with the highest occurrence frequency is 2.7.11.1 (non-specific serine/threonine protein kinase; 80 occurrences), followed by 3.6.4.13 (RNA helicase; 46 occurrences) and 3.4.19.12 (ubiquitinyl hydrolase 1; 23 occurrences).

Also the *P. sanguineus* transcriptome was annotated by mapping the transcripts onto the pathways reported in the Kyoto Encyclopedia of Genes and Genomes (KEGG) using the KAAS server. A total of 2,554 transcripts (34.9%) were annotated (Table S4).

Additionally, the *P. sanguineus* sequences were searched against the Cluster of Orthologous Groups of proteins (COG) database of the NCBI. A total of 2,468 (33.8%) transcripts were assigned to COG functional categories using the BLASTx algorithm with an E-value cutoff threshold of 10^−10^. Among 25 categories, the "General function prediction only" was the one receiving more hits (616), followed by "Amino acid transport and metabolism" (294), “Transcription (241), “Translation” (236) and "Carbohydrate transport and metabolism" (232) (Table S5).

The HMMSearch function from the HMMER package was used to compare the *P. sanguineus* translated transcriptome against the PFAM and TIGRFAM protein databases (Table S1). As previously observed in other basidiomycetes [12], most abundant matches included families associated to transmembrane transport (MFS transporter, ABC transporter and sugar transporter), oxidoreductase (Cytochrome P450, GMC oxidoreductase), hydrolase, signal transduction and nucleotide binding proteins (Table S6).

Finally, putative functions were manually assigned for 4,732 (64.8%) transcripts taking into consideration similarities of translated sequences against our customized database -including basidiomycetes protein sequences from the UniProt database and *T. versicolor* and *P. chrysosporium* sequences present in NCBI non-redundant protein database- and the presence of conserved protein domains, as well as EC number, GO terms, KEGG and COG assignations. All the remaining transcripts (2,551) showing no significant hits or inconsistent assignations were annotated as encoding hypothetical proteins (Table S1).

### Overview of gene expression with biotechnological relevance

**Figure 3:**
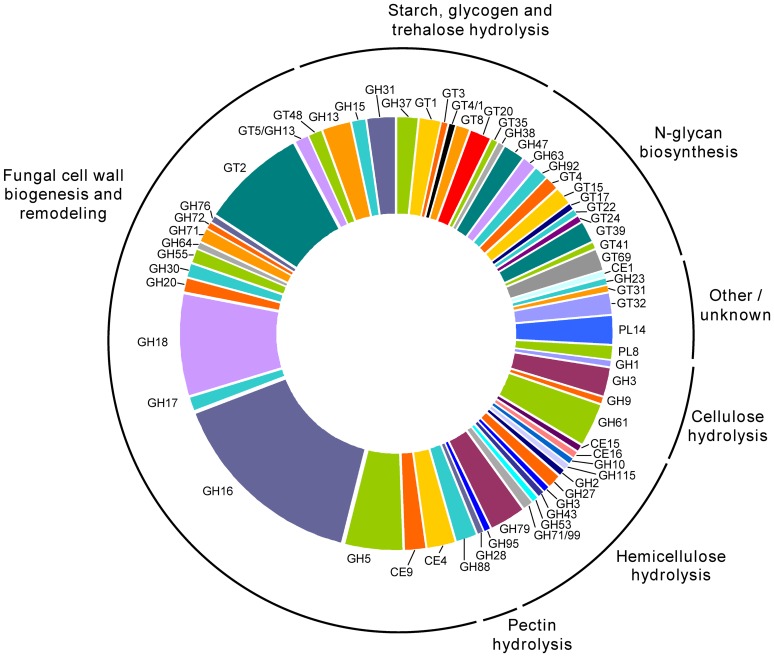
Distribution of *P. sanguineus* predicted CAZymes. Transcripts encoding putative carbohydrate active enzymes were assigned to seven functional categories according to their predicted function. GH: glycoside hydrolase, GT: glycosyltransferase, CE: carbohydrate esterase, PL: polysaccharide lyase.

**Enzymes related to carbohydrate metabolism.** Analysis of *P. sanguineus* transcriptome revealed 178 ORFs encoding predicted carbohydrate active enzymes (CAZy) distributed in 60 CAZy families. From these families, 35 were glycoside hydrolases (GH, 115 proteins), 18 glycosyltransferases (GT, 47 proteins), 5 carbohydrate esterases (CE, 10 proteins) and 2 polysaccharide lyases (PL, 6 proteins) (Table 2). Most of the identified transcripts encoded proteins belonging to CAZy families with predicted functions related to the synthesis and hydrolysis of β-1,3-glucans and chitin, thus reflecting the dynamism of cell wall biogenesis and remodeling in filamentous fungi, and their putative role in the initiation of autophagy processes triggered by nutrient starvation (Figure 3 and Table S7).

**Table 2:** Summary of predicted CAZymes in *P. sanguineus* transcriptome.

CAZyme Class	n^o^ of families	n^o^ of proteins
GH: Glycoside hydrolases	35	115
GT: Glycosyltransferases	18	47
CE: Carbohydrate esterases	5	10
PL: Polysaccharide lyases	2	6
**Total**	**60**	**178**

Despite the absence of any lignocellulosic substrate in the culture media, it was possible to detect transcripts encoding putative glycoside hydrolases involved in plant cell wall degradation, including cellulases (GH9 and GH61 families), β-glucosidases (GH1 and GH3 families), hemicellulases and pectinases (GH2 β-1,4-mannosidase, GH3 xylan 1,4-β-xylosidase, GH10 β-1,4-endoxylanase, GH27 α-galactosidase, GH28 rhamnogalacturonase, GH43 arabinanase, GH53 β-1,4-endogalactanase, GH79 β-glucuronidase, GH88 glucuronyl hydrolase, GH95 α-fucosidase, GH115 α-glucuronidase). Although their presence in all of the sequenced white-rot fungi genomes, no transcripts encoding any of the canonical endoglucanases (GH5 and GH12 families) or cellobiohydrolases (GH6 and GH7 families), were detected in *P. sanguineus* suggesting that their expression in this fungus is subjected to a tighter regulation than hemicellulases. As extensively shown in filamentous fungi [47], [48], transcripts of cellulases in white-rot fungi are upregulated in absence of glucose, by the release of carbon catabolite repression mechanisms [49], and also by the presence of a lignocellulosic substrate. Endoglucanase and cellobiohydrolase transcripts from *P. chrysosporium* and *P. carnosa* and to a lesser extent from *C. subvermispora* were demonstrated to be induced by the presence of a cellulosic or wood substrates [50], [51], [12], however many of them were also moderately upregulated in ligninolytic media. Thus, the apparent absence of transcripts encoding typical cellulases in the *P. sanguineus* transcriptome could be the result of the carbon catabolite repression due to the presence of traces of glucose at the time of harvesting, and the lack of a lignocellulosic inductor. As a consequence, a higher sequencing coverage than used in this study might be necessary to detect these low expressed transcripts in the conditions tested.

**Enzymes related to lignin hydrolysis and modification.** *Multicopper oxidases* **.** Four transcripts encoding enzymes belonging to multicopper oxidase (MCO) family were identified (Table 3). Both Psang02645 and Psang01483 translated ORFs corresponded to laccases (EC 1.10.3.2) previously characterized in *P. sanguineus* (GenBank ACZ37083, [52]; GenBank ACO51010, [53]). Although our assembly retrieved only partial sequences, amino acid identities were up to 99% and both included the conserved L3 and L4 signatures for HXH motifs [12], [54]. Since our fungal culture was supplemented with Cu^2+^ to induce laccase expression, and previous studies in *P. sanguineus* and *P. cinnabarinus* described only two different laccases [31], we assumed that these isoenzymes could be the only ones highly expressed in *P. sanguineus*. Psang02736 partial sequence encoded a putative protein showing an L2 signature different from the signature found in laccases and more similar to other polyporales MCOs, while Psang00791 corresponded to a canonical Fet3 ferroxidase related to iron homeostasis (Figure S1).

**Table 3:** *P. sanguineus* putative multicopper oxidases.

P. sanguineus ID^a^	Putative function	Blastx best hit description^b^	aa identity
Psang01483 (GAKI01001330)	Laccase	Laccase [Trametes sanguinea], (ACO51010)	98%
Psang02645 (GAKI01002490)	Laccase	Laccase [Trametes sanguinea], (ACZ37083)	99%
Psang02736 (GAKI01002581)	Multicopper oxidase	Multicopper oxidase [Dichomitus squalens LYAD-421 SS1], (EJF61736)	77%
Psang00791 (GAKI01000639)	Fet3 ferroxidase	Fet3 protein [Dichomitus squalens LYAD-421 SS1], (EJF63922)	86%

a Numbers in parentheses correspond to GenBank accession numbers for nucleotide sequences.

b Numbers in parentheses correspond to GenBank accession numbers for amino acid sequences.

*Peroxidases and related enzymes.* Five *P. sanguineus* transcripts encoded protein sequences homologous to known class II heme-peroxidases related to lignin degradation. Translated sequence alignment and identification of characteristic amino acid residues [55], [56] were used to classify them as manganese-dependent peroxidases (MnP, EC 1.11.1.13), lignin peroxidases (LiP, EC 1.11.1.14) or versatile peroxidases (VP, EC 1.11.1.16) (Table 4). Sequence Psang05490 was annotated as encoding a putative MnP since its translated ORF showed high amino acid identity (91%) with *Lenzites gibbosa* manganese peroxidase 3 (GenBank AEX01147) including a conserved E210 residue, which is part of the Mn(II) oxidation site. Psang05937 translated sequence, was classified as a putative LiP because of its homology with *P. cinnabarinus* lignin peroxidase 2 (GenBank ADK60911) and the presence of the conserved W171 catalytic residue. Sequences Psang06299, Psang05248 and Psang07066 encode proteins showing homologies with two different *T. versicolor* manganese-repressed peroxidases, (GenBank AAB63460, CAG32981) and to *T. versicolor* lignin peroxidase isoenzyme LP7 (GenBank CAA83147), respectively. Since the three proteins were recently described as probable versatile peroxidases [17], [55], *P. sanguineus* orthologues were annotated as such; however further studies will be necessary to characterize their function.

**Table 4:** *P. sanguineus* putative peroxidases.

P. sanguineus ID^a^	Putative function	Blastx best hit description^b^	aa identity
Psang05490 (GAKI01005323)	Manganese peroxidase	Manganese peroxidase 3 [Lenzites gibbosa], (AEX01147)	91%
Psang05937 (GAKI01005768)	Lignin peroxidase	Lignin peroxidase-like 2 [Trametes cinnabarina], (ADK60911)	97%
Psang06299 (GAKI01006130)	Versatile peroxidase	Manganese-repressed peroxidase [Trametes versicolor], (AAB63460)	83%
Psang05248 (GAKI01005082)	Versatile peroxidase	Manganese-repressed peroxidase [Trametes versicolor FP-101664 SS1], (EIW62513)	93%
Psang07066 (GAKI01006891)	Versatile peroxidase	Lignin peroxidase isozyme LP7 [Trametes versicolor], (CAA83147)	94%
Psang01533 (GAKI01001380)	Cytochrome c peroxidase	Cytochrome c peroxidase [Trametes versicolor FP-101664 SS1], (EIW63252)	84%
Psang00188 (GAKI01000131)	Chloroperoxidase	Chloroperoxidase [Trametes versicolor FP-101664 SS1], (EIW53420)	64%
Psang01942 (GAKI01001788)	Chloroperoxidase	Chloroperoxidase [Trametes versicolor FP-101664 SS1], (EIW61336)	71%
Psang06212 (GAKI01006043)	Chloroperoxidase	Chloroperoxidase-like protein [Dichomitus squalens LYAD-421 SS1], (EJF56466)	77%
Psang00278 (GAKI01000192)	Linoleate 8R-lipoxygenase	Linoleate diol synthase [Trametes versicolor FP-101664 SS1], (EIW58238)	74%
Psang00411 (GAKI01000287)	Linoleate 8R-lipoxygenase	Heme peroxidase [Trametes versicolor FP-101664 SS1], (EIW63895)	86%

a Numbers in parentheses correspond to GenBank accession numbers for nucleotide sequences.

b Numbers in parentheses correspond to GenBank accession numbers for amino acid sequences.

Other transcripts encoding predicted peroxidases included a cytochrome-*c* peroxidase (Psang01533), three heme-thiolate peroxidases (Psang00188, Psang01942, Psang06212), and two putative linoleate 8R-lipoxygenases sequences showing a conserved animal heme-peroxidase protein domain (Psang00278 and Psang00411).

Since hydrolysis of non-phenolic structures of lignin by MnPs has been demonstrated to be coupled to linoleic acid peroxidation [57] we investigated the possible expression of enzymes related to fatty acid metabolism. Three *P. sanguineus* translated sequences matched with fatty acid desaturases involved in the biosynthesis of linoleic acid. Psang00112 translated ORF showed 70% and 73% amino acid identity with the Δ12-fatty acid desaturases (EC 1.14.19.6) FAD2 identified in *P. chrysosporium* (GenBank ACJ26016) [58] and *C. subvermispora* (GenBank BAJ04705) [59], respectively. Psang01003 and Psang03572 both encoded putative Δ9-fatty acid desaturases (EC 1.14.19.1) and Psang01003 translated ORF showed 79% and 82% identities with *P. chrysosporium* and *C. subvermispora* Δ9-fatty acid desaturases ole1, respectively (GenBank BAJ04706 and GenBank BAJ04704) [59], while Psang03572 encoded a protein with 58% identity with a second Δ9-fatty acid desaturase identified in *C. subvermispora* (GenBank EMD32546) [12]. In the three cases the highest amino acid identity was observed to *T. versicolor* orthologues (GenBank EIW59140, EIW55447 and EIW64164): 83%, 89% and 67% for Psang00112, Psang01003 and Psang03572, respectively (Table S8).

*Extracellular hydrogen peroxide generation and iron homeostasis* **.** Processes involving the production of hydrogen peroxide are particularly important for lignin degradation, since it is required for the catalytic activity of peroxidases and the initial attack of lignin by hydroxyl radicals, generated through the Fenton reaction [60]. Analysis of *P. sanguineus* transcriptome revealed multiple transcripts encoding glucose-methanol-choline (GMC) oxidoreductases and copper radical oxidases potentially involved in generation of extracellular hydrogen peroxide, as well as enzymes involved in the generation of reduced iron. Fifteen *P. sanguineus* translated transcripts matched with reported GMC oxidoreductases and showed conserved related protein domains (Table 5). Both, Psang07044 and Psang01120 translated ORFs showed homologies (68% and 82%, respectively) with an aryl-alcohol oxidase-like protein (EC 1.1.3.7) from *T. versicolor* (GenBank EIW51595). Since there is no superposition between Psang07044 and Psang01120 sequences, they could represent parts of the same transcript, in which Psang0744 encodes the first 107 amino acids from the N-terminal region, including a putative signal secretion sequence, and Psang01120 encodes the 473 amino acids of C-terminal region. The ORF encoded by Psang01120 also included the conserved H502 and H546 residues involved in substrate binding and oxidation in aryl-alcohol oxidases [61]. Regarding the three aromatic residues involved in the regulation of substrate access to the binding site, Y92 and F397 are present in Psang01120 and in the *T. versicolor* orthologue; however the F501 is replaced by an arginine in both. Since substitutions at position 501 have shown to alter oxygen kinetics [62], further cloning and characterization of this enzyme will be necessary to confirm its biological function.

**Table 5:** *P. sanguineus* putative GMC oxidoreductases.

P. sanguineus ID^a^	Blastx best hit description^b^	aa identity	Conserved protein domains
Psang01120 (GAKI01000967)	Aryl-alcohol oxidase-like protein [Trametes versicolor FP-101664 SS1] (EIW51595)	82%	GMC_oxred_C[pfam05199], GMC oxidoreductase/BetA[COG2303], Choline dehydrogenase and related flavoproteins
Psang07044 (GAKI01006869)	Aryl-alcohol oxidase-like protein [Trametes versicolor FP-101664 SS1] (EIW51595)	68%	PRK02106[PRK02106], Choline dehydrogenase
Psang02094 (GAKI01001940)	Pyranose 2-oxidase [Trametes versicolor FP-101664 SS1] (EIW52665)	81%	Pyranose_ox[TIGR02462], Pyranose oxidase
Psang02251 (GAKI01002097)	Pyranose 2-oxidase [Trametes versicolor FP-101664 SS1] (EIW52665)	65%	Pyranose_ox[TIGR02462], Pyranose oxidase
Psang00492 (GAKI01000361)	Putative pyranose oxidase [Auricularia delicata TFB-10046 SS5] (EJD34922)	94%	Pyranose_ox[TIGR02462], Pyranose oxidase
Psang02237 (GAKI01002083)	Alcohol oxidase [Trametes versicolor FP-101664 SS1] (EIW52847)	83%	PRK02106[PRK02106], Choline dehydrogenase
Psang01295 (GAKI01001142)	Alcohol oxidase-like protein [Trametes versicolor FP-101664 SS1] (EIW56549)	70%	PRK02106[PRK02106], Choline dehydrogenase/BetA[COG2303], Choline dehydrogenase and related flavoproteins
Psang03086 (GAKI01002929)	Alcohol oxidase [Trametes versicolor FP-101664 SS1] (EIW56999)	69%	PRK02106[PRK02106], Choline dehydrogenase
Psang03470 (GAKI01003310)	Alcohol oxidase [Trametes versicolor FP-101664 SS1] (EIW62184)	93%	GMC_oxred_C[pfam05199], GMC oxidoreductase
Psang00518 (GAKI01000383)	Alcohol oxidase [Dichomitus squalens LYAD-421 SS1] (EJF60559)	58%	GMC_oxred_C[pfam05199], GMC oxidoreductase/BetA[COG2303], Choline dehydrogenase and related flavoproteins
Psang00419 (GAKI01000295)	Alcohol oxidase [Dichomitus squalens LYAD-421 SS1](EJF60559)	53%	
Psang02513 (GAKI01002359)	Alcohol oxidase [Trametes versicolor FP-101664 SS1] (EIW62184)	95%	PRK02106[PRK02106], Choline dehydrogenase/BetA[COG2303], Choline dehydrogenase and related flavoproteins
Psang00710 (GAKI01000559)	GMC oxidoreductase [Trametes versicolor FP-101664 SS1] (EIW62405)	77%	GMC_oxred_C[pfam05199], GMC oxidoreductase/BetA[COG2303], Choline dehydrogenase and related flavoproteins
Psang03627 (GAKI01003466)	GMC oxidoreductase [Trametes versicolor FP-101664 SS1] (EIW56548)	68%	PRK02106[PRK02106], Choline dehydrogenase/BetA[COG2303], Choline dehydrogenase and related flavoproteins
Psang07360 (GAKI01007174)	GMC oxidoreductase [Trametes versicolor FP-101664 SS1] (EIW54978)	68%	GMC_oxred_C[pfam05199], GMC oxidoreductase/PRK02106[PRK02106], Choline dehydrogenase

a Numbers in parentheses correspond to GenBank accession numbers for nucleotide sequences.

b Numbers in parentheses correspond to GenBank accession numbers for amino acid sequences.

Additionally, the three different ORFs encoded by Psang02094, Psang00492 and Psang02251 showed homologies with pyranose 2-oxidases (EC 1.1.3.10) from *T. versicolor* and *T. hirsuta* (Table 5). Psang02094 translated sequence showed the conserved H548 and N593 residues part of the active site, as well as the D452/F454/Y456 residues form the substrate recognition loop found in pyranose 2-oxidases [63]. Psang00492, encoding the N-terminal region of a putative pyranose 2-oxidase, showed the conserved H167 involved in the flavin cofactor covalent linkage [64] and T169 capable of forming H-bonds near the productive enzyme-substrate complex needed for efficient flavin reduction [65]. Finally, Psang02251, although encoding a partial sequence with homology (65%) to a pyranose 2-oxidase from *T. versicolor*, showed a H167R mutation. Cloning of the corresponding cDNA will be necessary to determine if it represents a real mutation or just a sequence error, since this could be due to a single base substitution (CAC x CGC).

Among the ten remaining sequences encoding ORFs with conserved domains related to GMC oxidoreductases, none of them showed conserved amino acids that could be used to classify them. Although seven of them showed high homology with sequences encoding putative alcohol (methanol) oxidases (EC 1.1.3.13) of *T. versicolor* and *D. squalens*, further analysis will be necessary to assign a proper function.

Although previously characterized in *P. cinnabarinus* [66], transcripts encoding cellobiose dehydrogenase (CDH, EC 1.1.99.18), a protein involved in Fe (III) reduction and cellulose degradation, were not detected in the conditions we tested. As postulated for cellulases and hemicellulases, the absence of CDH could be explained by the presence of traces of glucose, since the expression of the *T. versicolor* orthologue is also strongly regulated at transcriptional level by carbon catabolite repression mechanisms [67].

The sustained production of hydrogen peroxide by extracellular aryl-alcohol oxidases is achieved by a cyclic redox reaction involving the reduction by aryl-alcohol dehydrogenases (EC 1.1.1.90) of aryl-aldehydes to the corresponding alcohols [68]. Supporting the existence of aromatic aldehyde redox cycling in *P. sanguineus,* 26 transcripts encoding proteins of the aldo/keto reductase family were found, of which at least four were putative aryl-alcohol dehydrogenases. Thus, Psang01767 and Psang03332 translated sequences showed high amino acid identity (67% and 75%, respectively) with a previously characterized *P. chrysosporium* aryl-alcohol dehydrogenase (GenBank Q01752) [69] whereas Psang06157 and Psang04221-encoded proteins have *T. versicolor* orthologues annotated as aryl-alcohol dehydrogenases (GenBank EIW61065 and EIW61070).

An additional source of extracellular hydrogen peroxide is glyoxal oxidase, a copper radical oxidase (CRO). Putative *P. sanguineus* CROs were classified according to their identity degree with *P. chrysosporium* reported sequences [70] (Table 6). Psang00738 and Psang00288 translated sequences showed 60% and 65% amino acid identity with *P. chrysosporium* Cro1 and Cro2, respectively and conserved residues, part of the Cu-coordinating active site found in glyoxal oxidase (Y135, Y377, H378, and H471) and the cysteine (C70) conforming the radical redox site, were identified in both sequences. Psang00289 encodes an ORF of 196 amino acids showing 100% identity with translated Psang00288, except for a 61-amino acids region, suggesting a splicing variant, similar to the observed for the Cro2 splicing variant A, described in *P. chrysosporium* (GenBank ABD97059). Also, both Psang00288 and Psang00289 encode a 49-amino acid C-terminal extension, not present in *P. chrysosporium* orthologues but in a related *T. versicolor* DUF1929 domain-containing protein (GenBank EIW56122). Furthermore, Psang01858 and Psang06824 translated sequences matched with *P. chrysosporium* Cro3 and Cro4, respectively and only the protein encoded by Psang03463, showed high amino acid identity (71%) with a glyoxal oxidase, previously characterized in *P. chrysosporium* (GenBank AAA87594) [71].

**Table 6:** *P. sanguineus* putative copper radical oxidases.

P. sanguineus ID^a^	P. chrysosporium best hit^b^	aa identity	Conserved protein domains
Psang03463 (GAKI01003303)	Glyoxal oxidase (AAA87594)	71%	Glyoxal oxidase N-terminus[pfam07250]
Psang00738 (GAKI01000586)	Copper-radical oxidase 1 (ABD61572)	63%	DUF1929[pfam09118]/Glyoxal oxidase N-terminus[pfam07250]
Psang00288 (GAKI01000200)	Copper-radical oxidase 2 (ABD61573)	65%	DUF1929[pfam09118]/Glyoxal oxidase N-terminus[pfam07250]
Psang00289 (GAKI01000201)	Copper-radical oxidase 2 (variant A), (ABD97059)	56%	-
Psang01858 (GAKI01001704)	Copper-radical oxidase 3 (ABD61574)	85%	DUF1929[pfam09118]
Psang06824 (GAKI01006651)	Copper-radical oxidase 4 (ABD61575)	82%	-

a Numbers in parentheses correspond to GenBank accession numbers for nucleotide sequences.

b Numbers in parentheses correspond to GenBank accession numbers for amino acid sequences.

An extra supply of Fenton reagents, involving the quinone redox-cycling has been postulated in wood decay fungi [72]. Quinones that derive of breakdown of lignin or *de novo* synthesized by the fungus can then be converted to hydroquinones by quinone reductases, oxidized to semiquinones by laccases and auto-oxidized back to quinone to generate superoxide anion radical (O_2_^−^). Although O_2_^−^ cannot degrade lignin by itself, it can oxidize Mn (II) to Mn (III), generate H_2_O_2_ by dismutation, and reduce Fe (III) to Fe (II). The analysis of *P. sanguineus* transcriptome, revealed a transcript encoding a putative quinone reductase (Psang02478) which showed 73% amino acid identity with the 1,4-benzoquinone reductase characterized in *P. chrysosporium* (GenBank AAD21025) [73] and 67% amino acid identity with a NADH:quinone oxidoreductase reported in *G. trabeum* (GenBank AAL67859) [74], thus supporting the existence of a quinone redox-cycling in *P. sanguineus* similar to that already demonstrated in *P. cinnabarinus* [75].

Additional transcripts encoding proteins potentially involved in iron homeostasis were identified in *P. sanguineus* transcriptome. These include 5 iron reductases (Psang01201, Psang01840, Psang04167, Psang00819, and Psang00992), 9 iron permeases (Psang00836, Psang00862, Psang01172, Psang03545, Psang07406, Psang07480, Psang07518, Psang07523 and Psang07526), 2 CTR copper transporters (Psang01990 and Psang03439), and an ATX1-type copper chaperone, a key protein for copper acquisition by Fet3 (Psang06297) (Table S1).

**Figure 4:**
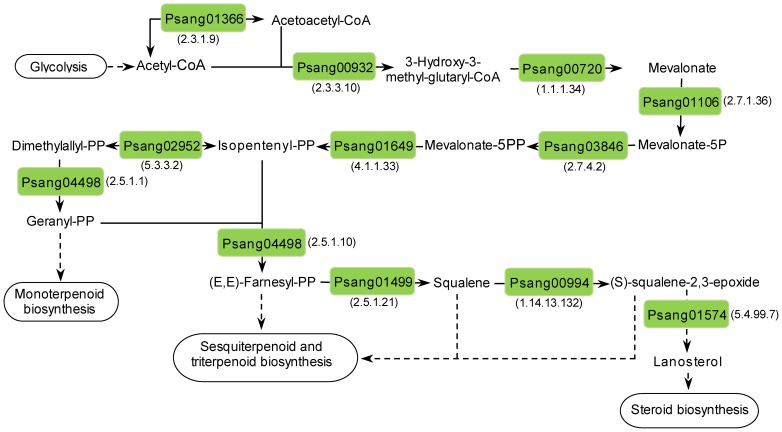
Reconstruction of terpenoid backbone biosynthesis pathway in *P. sanguineus*. Psang numbers inside boxes represent the IDs of transcripts encoding predicted enzymes involved in the biosynthesis of isopentenyl pyrophosphate via the mevalonate pathway, triterpenoid precursors and lanosterol. Numbers between brackets indicate the EC number of the corresponding enzyme. Dashed arrows indicate multiple steps.

**Terpenoid biosynthesis.** Fungi are important sources of bioactive secondary metabolites including various sesquiterpenes and triterpenes. Among these latter, ganoderic acids, showing anticancer, antiviral and hepatoprotective activity, were characterized in *G. lucidum* [76], [77]. All of the enzymes involved in the terpenoid backbone biosynthesis via the mevalonate pathway that were previously identified in *G. lucidum* [78], [79] have orthologues encoded by *P. sanguineus* transcripts (Table 7, Figure 4). We also identified transcripts encoding a putative squalene synthase (Psang01499), a putative squalene monooxygenase (Psang00994) and a putative lanosterol synthase (Psang01574), responsible for the biosynthesis of sesquiterpenoids and triterpenoids precursors and lanosterol, the precursor of steroids and ganoderic acids. Psang01106 encoded a fusion protein between an N-terminal cystathione beta-lyase (metC) and C-terminal mevalonate kinase (MVK). Though similar fusions have been previously observed in other basidiomycetes [79], their biological relevance remains unknown.

**Table 7:** *P. sanguineus* predicted genes involved in terpenoid biosynthesis.

P. sanguineus ID^a^	Predicted enzyme	EC number	G. lucidum ortholog^b^
Psang01366 (GAKI01001213)	Acetyl-CoA acetyltransferase (thiolase), AACT	2.3.1.9	G_lucidum_10003032
Psang00932 (GAKI01000780)	3-Hydroxy-3-methylglutaryl-CoA synthase, HMGS	2.3.3.10	G_lucidum_10008701
Psang00720 (GAKI01000569)	3-Hydroxy-3-methylglutaryl CoA reductase, HMGR	1.1.1.34	G_lucidum_10003589
Psang01106 (GAKI01000953)	Mevalonate kinase, MVK	2.7.1.36	G_lucidum_10009892
Psang03846 (GAKI01003685)	Phosphomevalonate kinase, MPK	2.7.4.2	G_lucidum_10010135
Psang01679 (GAKI01001525)	Diphosphomevalonate decarboxylase, MDV	4.1.1.33	G_lucidum_10005090
Psang02952 (GAKI01002796)	Isopentenyl-diphosphate isomerase, IDI	5.3.3.2	G_lucidum_10001705
Psang04498 (GAKI01004336)	(2E,6E)-Farnesyl diphosphate synthase, FPP	2.5.1.10	G_lucidum_10002724G_lucidum_10008471G_lucidum_10004225
Psang01499 (GAKI01001346)	Squalene syntase, SQS	2.5.1.21	G_lucidum_10005172
Psang00994 (GAKI01000842)	Squalene monooxygenase, SE	1.14.13.132	G_lucidum_10007072
Psang01574 (GAKI01001420)	Lanosterol synthase, LS	5.4.99.7	G_lucidum_10008645G_lucidum_10008646

a Numbers in parentheses correspond to GenBank accession numbers for nucleotide sequences.

b *G. lucidum* orthologs IDs are according to published in [79].

Additionally, transcripts encoding another 6 terpene synthases were found in *P. sanguineus*. Three of these showed homologies with terpene synthases described in *C. cinerea* [80]. Psang02278 translated ORF showed 73% identity with *C. cinerea* Cop3 (GenBank XP_001832925), characterized as an alfa-muurolene synthase (EC 4.2.3.125) and Psang02180 encodes a protein showing 60% identity with *C. cinerea* Cop2 (GenBank XP_001836556), and Cop1 (GenBank XP_001832573), both characterized as germacrene A synthases; whereas Psang01395 translated ORF showed a 46 to 47% identity with Cop1, Cop2 and Cop3. The other 3 translated sequences encoding putative terpene synthases, Psang04116, Psang02169 and Psang01353 have identities of 33% or less with *C. cinerea* enzymes, although all of them contained the conserved isoprenoid biosynthesis enzyme class 1 protein domain.

Also related to the biosynthesis of triterpenes and the metabolism of xenobiotics and lignin substructures, transcripts encoding 67 putative cytochrome P450 monooxygenases (EC 1.14.13.x, EC 1.14.14.x) are present in *P. sanguineus* transcriptome. Additionally, we identified sequences encoding 8 putative glutathione S-transferases (EC 2.5.1.18) and 3 epoxide hydrolases (EC 3.3.2.10) belonging to alpha/beta hydrolase protein family (Table S1), with potential in the biodegradation of many organic compounds by cytochrome P450 monoxygenases and the enantioselective biosynthesis of biologically active drugs [81].

## Discussion and Conclusions

Wood decay basidiomycetes are characterized by its ability to degrade lignocellulose through the biosynthesis of a complex set of extracellular hydrolases and oxidative enzymes. They are broadly divided into three groups according to their strategy to degrade lignin in order to allow the access of hydrolytic enzymes to plant cell wall polysaccharides. While brown-rot fungi and the less studied soft-rot fungi perform partial depolymerization of lignin, white-rot fungi are the only microorganisms described to date capable of its complete mineralization. In white-rot fungi the expression of ligninolytic enzymes is generally triggered by nutrient depletion during secondary metabolism, although differential responses to C/N ratios and even to the presence of a lignocellulosic substrate have been observed among individual enzymes and fungal species [12], [50], [82]–[86]. Additionally, expression of laccases and MnPs have been shown to be induced by the presence of copper and/or manganese in *P. ostreatus* [33], *T. versicolor* [34], *T. trogii* [35] *Phlebia radiata* [86], *C. subvermispora* [87], and *Coriolopsis rigida* [88]. Furthermore, cis-acting elements related to metals and xenobiotics response mechanisms, and temperature shock or oxidative stress responses have been identified in the promoter regions of fungal laccases and class II heme-peroxidases (reviewed in [89]), supporting their putative role not only in wood decomposition but as detoxifying enzymes in response to environmental stresses.

In order to identify the transcripts encoding enzymes involved in lignin degradation in *P. sanguineus*, we performed the sequence of the transcriptome of this fungus grown at stationary phase, and in presence of CuSO_4_. According to this, we detected two transcripts encoding previously characterized laccases, five encoding putative class II heme-peroxidases and many transcripts encoding enzymes related to the generation of peroxide and free radicals involved in the initial attack of lignin. Although our study was not designed to perform a differential expression analysis, comparison with previous transcriptomic and extracellular proteomic studies performed in white-rot fungi showed this pattern of expression is consisting with the observed in nutrient-limiting conditions. Extracellular proteomic analysis by mass spectrometry (LC-MS/MS) of *P. chrysosporium* grown in ligninolytic media (carbon and nitrogen-limited) showed the expression of a glyoxal oxidase and from 5 to 8 class II peroxidases of the 15 genes predicted by genomic analysis [50], [84], [90], [91]. Proteomic studies in *T. versicolor* grown in tomato juice supplemented with CuSO_4_ and MnCl_2_ [92] and in *T. trogii* grown in a minimal media [93] detected peptides corresponding to 2 to 8 class II heme-peroxidases, 2 laccases and a glyoxal oxidase but also for GMC oxidoreductases including one pyranose 2-oxidase and one aryl-alcohol oxidase in both *T*. *versicolor* and *T. trogii*, and two methanol oxidases in the latter. Additional micro array-based transcriptional analysis performed in *P. chrysosporium* have shown that the genes encoding enzymes related to lignin depolymerization are mainly upregulated in nutrient-limited media and generally not highly induced by the presence of lignocellulosic substrates [50], [85]. However, comparative transcriptional studies in *C. subvermispora* and *P. carnosa* showed the upregulation of genes encoding class II heme-peroxidases and enzymes related to redox cycling processes when these fungi are grown in wood substrates relative to glucose [12], [51].

As extensively shown in ascomycetes [47], [48] expression of cellulases in wood decay basidiomycetes seem to be strongly regulated by carbon catabolite repression mechanisms mediated by CreA (cAMP mediated glucose repression) and also by the presence of a wood or cellulosic substrate. Most of the genes encoding endoglucanases (GH5, GH12), cellobiohydrolases (GH6, GH7), and GH61 cellulases have been shown to be strongly upregulated in *P. crhysosporium* and in *P. carnosa* grown in wood as sole carbon source relative to glucose, whereas only three canonical cellulases of eight gene models were significantly upregulated in *C. subvermispora* in presence of lignocellulosic substrates [12], [50], [51], [85]. Corresponding peptides were detected by LC-MS/MS in similar culture conditions for these fungi [12], [50], [94] and also for *A. delicata*, *T. versicolor*, *S. squalens*, *S. hirsutum*, and *P*. *strigosozonata* grown in aspen [17]. In our present study of *P. sanguineus* transcriptome we failed to detect the expression of any of the canonical cellulases, and only transcripts encoding two families of GHs with potential celullolytic activity were detected (GH9 and GH61). However, only the predicted GH9 endo-1,4-β-glucanase could be strictly assigned as a cellulase, since GH61 members has been recently redefined as copper-dependent lytic polysaccharide monooxygenases, implied in the oxidative cleavage of cellulose [95]. This apparent absence of transcripts encoding cellulases in *P. sanguineus* could be explained by the fact that no lignocellulosic substrate was used for fungal grown and also by the presence of traces of glucose at time of harvesting. This is also supported because we were unable to detect transcripts for cellobiose dehydrogenase, a flavooxidase that is proposed to contribute to peroxide generation, but mainly to enhance oxidative cellulose depolymerization and whose expression has been shown to be induced by lignocellulosic substrates [12], [50] and strongly repressed by glucose [67].

Another component of plant cell walls, hemicellulose, is a branched polymer consisting of a more heterogeneous assembly of monosaccharides and linkages than cellulose, thus a more complex set of enzymes is necessary for its hydrolysis. Although hemicellulose composition and structure depends on the plant source, studies performed in *P. carnosa* and *P. chrysosporium* grown in diverse wood and lignocellulosic substrates have shown similar pools of expressed hemicellulases and pectinases [50], [51], [90], [91], [94], suggesting that differential hydrolysis is regulated by modifying the relative abundance of the essentially equal profile of enzymes. Extracellular proteomic studies have commonly found peptides corresponding to β-1,4-mannosidases (GH2 family), β-xylanases (GH10 family), polygalacturonases (GH28 family), α-galactosidases (GH27 family), β-mannanases (GH5 families), arabinosidases (GH43 family) and acetyl xylan esterases (CE1 family) in the presence of a lignocellulosic substrate [50], [90], [94], but also for GH10, GH28 families in ligninolytic conditions [84], [90], [91].

Transcripts potentially encoding many of these hemicellulases were detected in our analysis of *P. sanguineus* transcriptome including members of mentioned common families (GH2, GH10, GH27, GH28, GH43) and also GH3 β-xylosidase, GH53 β-1,4-endogalactanase, GH79 β-glucuronidase, GH88 glucuronyl hydrolase, GH95 α-fucosidase, GH115 α-glucuronidase and CE15, CE16 debranching esterases, showing that this fungus expresses a basal set of hemicellulases even in the absence of a lignocellulosic inductor.

This pattern of expression in which hemicellulases, pectinases and enzymes related to the hydrolysis of lignin are constitutively expressed or induced under nutrient starvation while cellulases are differentially expressed and subjected to a more tight regulation, suggests a selective strategy for lignin and hemicellulose degradation in advance to cellulose; in contraposition to the second pattern of wood decay found in white-rot fungi in which all the components of plant cell walls are degraded simultaneously. This is consistent with previous delignification studies performed in *P. taeda* wood chips, in which treatment with *P. sanguineus* BAFC 2126 resulted in notable structural changes of lignin and hemicellulose over cellulose, as revealed from ^13^C CP-MAS NMR spectra [32]. On the other hand, studies on delignification of *Eucalyptus grandis* using a different strain, *P. sanguineus* UEC2050, have shown a simultaneous pattern of wood decay [96]. Although these results suggest that *P. sanguineus* may shift between delignification patterns depending on the wood it grows on, it can also be a consequence of different incubation times evaluated in each study (14 days for the first study and 2 to 4 months for the second), since selective degradation could slowly progress to a simultaneous-like pattern as wood hydrolysis progress.

Selective strategies in which lignin is removed preferentially to cellulose are important for applications in pulping industry and consequently there is great interest in understanding how they are achieved at molecular level. Although further studies will be necessary, our gene expression analysis in *P. sanguineus* suggests an increase in the ligninolytic potential relative to the cellulolytic capability. This is similar to the observed in comparative genomic and transcriptomic studies in the selective *C. subvermispora* and *P. carnosa* against the simultaneous degrader *P. chrysosporium*, supporting the potential of *P. sanguineus* for its evaluation in biopulping processes.

A striking characteristic of the basidiomycetes, especially of polyporales, is their ability to synthesize secondary metabolites of medical and industrial interest, including compounds with antiviral, anti-inflammatory, antimicrobial or anticancer activities, as well as antioxidants, aromas and flavors [97]. Pharmacologically active triterpenoids and sterols have been identified in *Piptoporus betulinus* [98], *Inonotus obliquus* [99], *Fomitopsis pinicola* [100], *W. cocos* [101], *Antrodia camphorata* [102], *Daedalea dickisii* [103], *Ganoderma applanatum* [104], and *G. lucidum* [76], [77], [105] among many others, however the detailed biosynthesis pathways in fungi are still under study. As previously reported in *G. lucidum* genomic studies [78], [79] exploration of *P. sanguineus* transcriptome allowed the identification of the transcripts encoding all the enzymes involved in terpenoid backbone biosynthesis pathway and also various terpene synthases related to the biosynthesis of important sesquiterpenoids, triterpenoids and sterols precursors.

Additionally we identified many transcripts encoding cytochrome P450 monooxygenases and glutathione S-transferases with potential in the biodegradation of xenobiotics and detoxification of lignin degradation products, as well as transcripts encoding putative epoxide hydrolases with potential for the enantioselective biosynthesis of biologically active drugs; showing the potential of *P. sanguineus* as a source of bioactive compounds and enzymes for the industry.

This paper presents the first sequencing and analysis of the transcriptome of *P. sanguineus* grown at stationary phase in presence of Cu^2+^. From the assembled 7,303 transcripts, putative functions were manually assigned for 4,732 by assessing translated sequences homologies and presence of conserved protein domains, allowing the identification of many transcripts encoding enzymes with biotechnological potential no previously reported in *P. sanguineus*. Due to the complexity of the wood decay process, which involves many enzymes with diverse activities, further studies are needed to fully understand the biochemical mechanisms that control this process in order to facilitate the selection of enzymes and fungal strains for specific industrial applications. Additionally, the metabolic pathways and enzymes involved in the biosynthesis of secondary metabolites in basidiomycetes are poorly studied and much work is necessary to identify and characterize the activities with potential application for organic synthesis and production of high added-value compounds.

The availability of this first version of the transcriptome of *P. sanguineus* may facilitate the analysis and annotation of additional sequencing projects and provide a tool for the study of metabolic pathways and the cloning and characterization of enzymes of biotechnological interest.

## Supporting Information

Figure S1 **Analysis of signatures for HXH motifs in** ***P. sanguineus*** **putative MCOs**. Tv: *T. versicolor*, Ds: *D. squalens*, Cs: *C. subvermispora*, Pc: *P. chrysosporium*, Pp: *P. placenta.* Fet3 proteins: Tv GenBank EIW55589, Ds GenBank EJF63922, Cs GenBank EMD34889, Pc GenBank ABE60664, Pp GenBank XP_002469890. MCOs: Tv GenBank EIW53804, Ds GenBank EJF61736, Cs GenBank EMD36964, Pp GenBank XP_002473277, Pc mco1: GenBank AAO42609, Pc mco2: GenBank AAS21659, Pc mco3: AAS21662, GenBank, Pc mco4: GenBank AAS21669. Shadowed letters indicates differences from the laccase consensus signature on top. Colored letters denotes differences in MCOs (blue) and Fet3 proteins (red) with *P. sanguineus* predicted sequences Psang02736 and Psang00791, respectively. An X in the signature represents an undefined residue while the multiple letters within brackets represent a partially conserved residue.
(PDF)

Table S1 ***P. sanguineus*** **transcripts.** List of IDs and functional annotation for the 7,303 transcripts identified in *P. sanguineus* grown in Cu^2+^.
(XLS)

Table S2 **Homologies of** ***P. sanguineus*** **assembly with** ***Pycnoporus*** **sequences annotated at NCBI database.**
(XLS)

Table S3 **Gene Ontology annotation.** List of GO terms assigned to 3,240 *P. sanguineus* transcripts using Blast2GO.
(XLS)

Table S4 **KEEG orthologies annotation.** List of KEEG orthology numbers assigned to 2,554 *P. sanguineus* transcripts using KAAS server.
(XLS)

Table S5 **COG annotation.** List of COG functional categories assigned to 2,468 *P. sanguineus* transcripts.
(XLS)

Table S6 **List of 50 most frequent PFAM domains in** ***P. sanguineus*** **transcriptome.**
(PDF)

Table S7 **Assignation of putative functions to predicted** ***P. sanguineus*** **CAZy families.**
(XLSX)

Table S8 ***P. sanguineus*** **putative fatty acid desaturases involved in the biosynthesis of linoleic acid.**
(PDF)
