# Supplementary material for: A First Insight into Pycnoporus sanguineus BAFC 2126 Transcriptome
Source: PLoS One. 2013 Dec 2;8(12):e81033. doi: 10.1371/journal.pone.0081033 (PMC3846667; doi:10.1371/journal.pone.0081033)
Supplement: Figure S1 — Analysis of signatures for HXH motifs in P. sanguineus putative MCOs. Tv: T. versicolor, Ds: D. squalens, Cs: C. subvermispora, Pc: P. chrysosporium, Pp: P. placenta. Fet3 proteins: Tv GenBank EIW55589, Ds GenBank EJF63922, Cs GenBank EMD34889, Pc GenBank ABE60664, Pp GenBank XP_002469890. MCOs: Tv GenBank EIW53804, Ds GenBank EJF61736, Cs GenBank EMD36964, Pp GenBank XP_002473277, Pc mco1: GenBank AAO42609, Pc mco2: GenBank AAS21659, Pc mco3: AAS21662, GenBank, Pc mco4: GenBank AAS21669. Shadowed letters indicates differences from the laccase consensus signature on top. Colored letters denotes differences in MCOs (blue) and Fet3 proteins (red) with P. sanguineus predicted sequences Psang02736 and Psang00791, respectively. An X in the signature represents an undefined residue while the multiple letters within brackets represent a partially conserved residue. (PDF) [file pone.0081033.s001.pdf]

	L1	L2
	HWHGxxxxxxxxxDGxxxxxQCPI	GTxWYHSHxxxQYCDGLxGx[FLIM]
**Psang02736**	**HWHGLFQRGTNYYDGTAAITQCGI**	**GSTWWHAHAGTQYTDGITGAF**
Tv mco	HWHGLFQRNTNFYDGTLAITQCGI	GSTWWHAHAGTQYTDGITGAL
Ds mco	HWHGLFQRGTNFYDGTDAITQCGI	GSTWWHAHYMTQYTDGIVGAF
Cs mco	HWHGLYQRGTNYYDGTAAITQCGI	GSTWWHAHYSTQYTDGITGAL
Pp mco	HWHGLYQNGTNYYDGTHAVTQCGI	GSTWWHAHADTQYTDGVVGAL
Pc mco1	HWHGIPQNGTAYYDGTAGITECGI	GTTWWHSHYDTQYTDGVTGAL
Pc mco2	HWHGLFQNQTNYYDGTAGITECGI	GTTWWHAHYSTQYTDGITGAL
Pc mco3	HWHGLFQNGTNYYDGTAAITECGI	GTTWWHAHYSTQYTDGITGAL
Pc mco4	HWHGLYQNSTNYYDGTAGVTECGI	GTTWWHAHYDTQYTDGVTGAL
**Psang00791**	**HHHGMYFNSTSWMDGAVGVSQCGI**	**GTYWVHSHAKGQYVDGLRGPV**
Tv Fet3	HHHGMFFNSTSWMDGAVGVSQCGI	GTYWVHAHASGQYVDGLRAPV
Ds Fet3	HHHGMFFNSTSWMDGAVGVSQCGT	GTYWIHSHASGQYVDGLRAPV
Cs Fet3	HHHGMFFNSTSWMDGALGVSQCGI	GTYWWHAHAKGQYVNGLRAPL
Pc Fet3	HHHGMFFNSTSWMDGALAISQCGV	GTYWVHSHASGQYVDGLRAPV
Pp Fet3	HHHGMFFNSTSWMDGAMGVSQCGI	GTYWVHAHSTGQYVDGLRSPV

	L3	L4
	HPxHLHGH	G[PA]Wx[LFV]HCHI[DAE]xHxxxG[LMF]xxx[LFM]
**Psang02645**	**HPFHLHGH**	**G P WF L HCH**
**Psang01483**	**HPFHLHGH**	**G P WF L HCHI D FHLEAG F AIV F**
Tv mco	HPFHLHGH	G F WA F HCHI Q WHMAAG L LFQ V
Ds mco	HPFHLHGQ	G F WA F HCHI Q WHMSAG L LFQ L
Cs mco	HPFHLHGH	G Y WA F HCHI Q WHMAAG L LFQ F
Pc mco1	HPFHLHGY	G A WT L HCHI S WHMSAG L LMQ F
Pc mco2	HPFHLHGH	G L WA F HCHL A WHMAAG L LMQ I
Pc mco3	HPFHLHGH	G L WA F HCHL A WHMAAG M LMQ V
Pc mco4	HPFHLHGH	G I WT L HCHI A WHMAAG L MMQ I
Pp mco	HPFHLHGY	G Y WT F HCHI Q WHMSAG L LFQ F
**Psang00791**	**HPFHLHGH**	**G V WF F HCHI E WHLEVG L AVT F**
Tv Fet3	HPFHLHGH	G A WF F HCHI E WHLEVG L AVT F
Ds Fet3	HPFHLHGH	G A WF F HCHI E WHLEVG L AVQ F
Cs Fet3	HPFHLHGH	G V WF F HCHI E WHLEVG L AVQ F
Pc Fet3	HPFHLHGH	G A WI F HCHI E WHLQAG L AVT F
Pp Fet3	HPFHLHGH	G V WF F HCHI E WHLEVG L AIQ L

Figure S1. **Analysis of signatures for HXH motifs in *P. sanguineus* putative MCOs**. Tv: *T. versicolor*, Ds: *D. squalens*, Cs: *C. subvermispora*, Pc: *P. chrysosporium*, Pp: *P. placenta.* Fet3 proteins: Tv GenBank EIW55589, Ds GenBank EJF63922, Cs GenBank EMD34889, Pc GenBank ABE60664, Pp GenBank XP002469890. MCOs: Tv GenBank EIW53804, Ds GenBank EJF61736, Cs GenBank EMD36964, Pp GenBank XP_002473277, Pc mco1: GenBank AAO42609, Pc mco2: GenBank AAS21659, Pc mco3: AAS21662, GenBank, Pc mco4: GenBank AAS21669. Shadowed letters indicates differences from the laccase consensus signature on top. Colored letters denotes differences in MCOs (blue) and Fet3 proteins (red) with *P. sanguineus* predicted sequences Psang02736 and Psang00791, respectively. An X in the signature represents an undefined residue while the multiple letters within brackets represent a partially conserved residue.
