# Supplementary material for: A First Insight into Pycnoporus sanguineus BAFC 2126 Transcriptome
Source: PLoS One. 2013 Dec 2;8(12):e81033. doi: 10.1371/journal.pone.0081033 (PMC3846667; doi:10.1371/journal.pone.0081033)
Supplement: Table S6 — List of 50 most frequent PFAM domains in P. sanguineus transcriptome. (PDF) [file pone.0081033.s007.pdf]

Table S6. List of 50 most frequent PFAM domains in *P sanguineus* transcriptome

Counts	Pfam description	Pfam ID
88	Protein kinase domain Pkinase	PF00069
65	WD domain, G-beta repeat WD40	PF00400
56	Cytochrome P450	PF00067
39	Major Facilitator Superfamily MFS_1	PF07690
38	RNA recognition motif. (a.k.a. RRM, RBD, or RNP domain)	PF00076
31	Aldo/keto reductase family	PF00248
29	Mitochondrial carrier protein	PF00153
26	short chain dehydrogenase	PF00106
25	ABC transporter	PF00005
22	Eukaryotic aspartyl protease	PF00026
22	Sugar (and other) transporter (Sugar_tr)	PF00083
21	Helicase conserved C-terminal domain	PF00271
21	Ras family	PF00071
19	Alpha/beta hydrolase family	PF12695
19	Zinc-binding dehydrogenase	PF00107
18	Core histone H2A/H2B/H3/H4	PF00125
18	Fungal hydrophobin	PF01185
18	Fungal Zn(2)-Cys(6) binuclear cluster domain	PF00172
17	Fungal specific transcription factor domain	PF04082
16	Aminotransferase class I and II	PF00155
16	FAD binding domain	PF01494
16	GMC oxidoreductase	PF00732
15	AMP-binding enzyme	PF00501
15	DEAD/DEAH box helicase	PF00270
14	Aldehyde dehydrogenase family	PF00171
14	Proteasome subunit	PF00227
13	G-protein alpha subunit	PF00503
13	Ring finger domain	PF13639
13	Tetratricopeptide repeat	PF13428
12	ATPase family associated with various cellular activities (AAA)	PF00004
11	Amino acid permease	PF00324
11	Glycosyl hydrolases family 18	PF00704
11	HEAT repeat	PF02985
11	KH domain	PF00013
11	Serine carboxypeptidase	PF00450
11	Ubiquitin carboxyl-terminal hydrolase	PF00443
11	Ubiquitin-conjugating enzyme	PF00179
10	Glycosyl hydrolases family 16	PF00722
10	Hsp70 protein	PF00012
9	Alcohol dehydrogenase GroES-like domain	PF08240
9	F-box-like	PF12937

9	HAD ATPase, P-type, family IC	TIGR01494
9	Helix-loop-helix DNA-binding domain	PF00010
9	Pro-kumamolisin, activation domain	PF09286
9	RhoGAP domain	PF00620
9	Ser-Thr-rich glycosyl-phosphatidyl-inositol-anchored membrane family	PF10342
9	TCP-1/cpn60 chaperonin family	PF00118
9	TPR repeat	PF13414
9	Zinc finger C-x8-C-x5-C-x3-H type (and similar)	PF00642
8	Acyl-CoA dehydrogenase, C-terminal domain	PF00441
