# Supplementary material for: A First Insight into Pycnoporus sanguineus BAFC 2126 Transcriptome
Source: PLoS One. 2013 Dec 2;8(12):e81033. doi: 10.1371/journal.pone.0081033 (PMC3846667; doi:10.1371/journal.pone.0081033)
Supplement: Table S8 — P. sanguineus putative fatty acid desaturases involved in the biosynthesis of linoleic acid. (PDF) [file pone.0081033.s009.pdf]

Table S8. ***P. sanguineus*** **putative fatty acid desaturases involved in the biosynthesis of linoleic acid**

P. sanguineus **ID**[a]	**Putative function**	**Orthologue**[b,c]		
		P. chrysosporium	***C. subvermispora***	***T. versicolor***
Psang00112 (GAKI01000088)	Δ-12 fatty acid desaturase	Δ -12 fatty acid desaturase FAD2 (ACJ26016), [70%]	Δ -12 fatty acid desaturase FAD2 (BAJ04705), [73%]	Δ -12 fatty acid desaturase (EIW59140), [83%]
Psang01003 (GAKI01000851)	Δ -9 fatty acid desaturase	Δ -9 fatty acid desaturase ole1 (BAJ04706), [79%]	Δ -9 fatty acid desaturase ole1 (BAJ04704), [82%]	Δ -9 fatty acid desaturase (EIW55447), [89%]
Psang03572 (GAKI01003411)	Δ -9 fatty acid desaturase	-	hypothetical protein CERSUDRAFT_87875 (EMD325469, [58%]	hypothetical protein TRAVEDRAFT_158133 (EIW64164), [67%]

[a] *Numbers in parentheses correspond to GenBank accession numbers for nucleotide sequences*

[b] *Numbers in parentheses correspond to GenBank accession numbers for amino acid sequences*

[c] *Number between brackets represents % of amino acid identities*
